# Targeting envelope lipids with 2,6‑di‑*O*‑methyl‑3‑acetyl‑β‑cyclodextrin impairs infectivity of SARS‑CoV‑2 and Japanese encephalitis virus

**DOI:** 10.1128/jvi.01357-25

**Published:** 2025-12-04

**Authors:** Daichi Kusakari, Naoki Kishimoto, Kazuyuki Oshiro, Yui Udeda, Nobutoki Takamune, Keiichi Motoyama, Shogo Misumi

**Affiliations:** 1 Department of Environmental and Molecular Health Sciences, Graduate School of Pharmaceutical Sciences, Kumamoto University 13205, Kumamoto, Japan; 2 Department of Physical Pharmaceutics, Graduate School of Pharmaceutical Sciences, Kumamoto University 13205, Kumamoto, Japan; University of North Carolina at Chapel Hill, Chapel Hill, North Carolina, USA

**Keywords:** SARS-CoV-2, 2,6-di-O-methyl-3-acetyl-β-cyclodextrin, aminophospholipids, effective antiviral strategies

## Abstract

Until pathogen-specific treatments and vaccines are available, early containment and prevention of further spread are essential. Here, we evaluated the antiviral activity of cyclodextrin (CyD) derivatives against enveloped RNA viruses, including SARS-CoV-2 and Japanese encephalitis virus (JEV). Among the 19 derivatives tested, 2,6-di-*O*-methyl-3-acetyl-β-cyclodextrin (DMA-β-CyD) exhibited the most potent antiviral activity against SARS-CoV-2 Wuhan, Beta, and Delta variants and demonstrated antiviral activity against the Wuhan variant (IC_50_ = 0.28 mM, CC_50_ ≥ 100 mM, selectivity index (SI) ≥ 357). Pretreatment of viral particles with DMA-β-CyD significantly reduced infectivity (by 2.25–2.67 log₁₀ units after 180 min of treatment) without affecting spike protein-ACE2 binding. DMA-β-CyD attenuated the procoagulant activity mediated by virion-associated aminophospholipids via interaction with the viral envelope. This effect was suppressed by 1-adamantanecarboxylic acid, suggesting that the inclusion of the target molecule of DMA-β-CyD is involved. Furthermore, DMA-β-CyD disrupted SARS-CoV-2 particles and reduced entry efficiency. Although the JEV-associated procoagulant activity was weaker than the SARS-CoV-2-associated procoagulant activity at 37°C, DMA-β-CyD recognized virion-associated aminophospholipids exposed on the surface of JEV particles. Against JEV, DMA-β-CyD exhibited comparable antiviral activity (IC_50_ = 0.84 mM, CC_50_ ≥ 100 mM, SI ≥119). These findings demonstrate that DMA-β-CyD exerts antiviral effects against both SARS-CoV-2 and JEV by targeting envelope aminophospholipids, which supports lipid-targeting strategies as a promising basis for broad-spectrum antiviral development.

**IMPORTANCE:**

The SARS-CoV-2 pandemic has highlighted the urgent need for effective antiviral strategies that can interrupt viral transmission before pathogen-specific vaccines or therapeutic interventions become available. In this study, we demonstrate that DMA-β-CyD is the first compound shown to reduce the infectivity of SARS-CoV-2 and Japanese encephalitis virus (JEV) particles by interacting with aminophospholipids, such as phosphatidylserine (PS). By exploiting differences in lipid composition between viral envelopes and host cell membranes, DMA-β-CyD provides a novel, lipid-targeting antiviral mechanism and may serve as a valuable platform for addressing infections caused by emerging and drug-resistant viral pathogens. This study underscores the therapeutic potential of disrupting envelope lipids as a broad-spectrum antiviral strategy.

## INTRODUCTION

The novel coronavirus (SARS-CoV-2) was first identified at the end of 2019 and rapidly disseminated worldwide, precipitating an unparalleled pandemic. In response, a novel modality, mRNA vaccine technology, was introduced, enabling a significant reduction in the time required for vaccine development and production (1). However, during the initial stages of the SARS-CoV-2 outbreak, there were no available vaccines or therapeutic drugs, and the sole recourse was to thoroughly implement standard precautions and take measures appropriate for the infection route. This scenario highlighted the necessity for the development of novel public health strategies aimed at limiting the transmission of infection during the initial stages of an unpredictable pandemic caused by an emerging virus.

Implementing effective strategies to inhibit the replication of emerging enveloped viruses, including novel coronaviruses, in the early stages of infection has proven useful in reducing the spread of infection. The interaction of viral spike proteins with receptors expressed on target cells is a common molecular mechanism of viral attachment and is a prominent area of research. The results of previous studies on viruses belonging to the same family can provide useful insights for the development of vaccines and antiviral drugs for new epidemics. Conversely, when a virus acquires a significant mutation that alters the epitope of the spike protein, new research and development is required. Thus, it is imperative to have effective antiviral strategies ready for multiple classes of viruses. In general, interfering with the lipid envelope is an effective virucidal strategy targeting enveloped viruses, and many studies have shown that substances such as ethanol and cetylpyridinium chloride can disrupt the envelope, thereby inactivating these pathogens (2). Moreover, greater specificity may be attained by targeting phospholipids, such as phosphatidylserine (PS) and phosphatidylethanolamine, that are effectively incorporated into the viral envelope of SARS-CoV-2 (3). Some viruses have acquired PS-enriched envelopes by exploiting the specificity of the subcellular localization of PS. For example, viruses belonging to the *Coronaviridae* family acquire an envelope with accumulated PS on the outer membrane by budding into the lumenal side of the endoplasmic reticulum (4). As a result, PS on the surfaces of SARS-CoV-2 viral particles binds to PS receptors (e.g., AXL, TIM-1, and TIM-4) on host cells and promotes viral infection (5). Similarly, the following viruses incorporate PS into their lipid envelope to facilitate entry via the host PS receptor. Vaccinia viruses, which belong to the *Poxviridae* family, form a specific endoplasmic-reticulum-derived membrane structure and bud on its lumenal side, thereby acquiring an envelope in which PS is accumulated (6). Dengue virus and Japanese encephalitis virus (JEV), which belong to the *Flaviviridae* family, and chikungunya virus, which belongs to the *Togaviridae* family, acquire a plasma-membrane-derived envelope with PS accumulated on the outer membrane and release viruses from the plasma membrane when apoptosis is induced in infected cells (7–10). It is noteworthy that the Ebola virus, which belongs to the *Filoviridae* family, has developed a strategy to facilitate the incorporation of PS into the viral envelope. This involves the colocalization of the activated scramblase XKR8 with the Ebola virus matrix (VP40) and glycoprotein (GP) (11). Therefore, the development of antibodies and therapeutics targeting PS may provide a means of limiting the spread of infection in the early stages of a pandemic and the transmission of emerging viruses.

Strategies that target PS, which is exposed on the lipid envelope of the viral surface, with the objective of blocking the interaction of PS on the viral surface with the host PS receptor, may prove an effective means of minimizing the spread of infection during the early stages of a pandemic. In a study conducted by Soares et al., guinea pigs infected with the Pichinde virus, in which PS is incorporated into the surfaces of the viral particles, were treated with bavituximab 6-7 days after infection (12). The results demonstrated that 50% of the animals survived, and the virus was eliminated by day 135 (12). The bavituximab treatment was shown to remove infectious viruses from the bloodstream as well as induce the antibody-dependent cytotoxicity of virus-infected cells (12). Furthermore, it is believed that bavituximab blocks PS exposure on viral surfaces and infected cells, preventing the virus from evading immune recognition (12, 13). Song et al. developed a biological agent, sTIM1dMLDR801, which can specifically inhibit the PS-dependent viral entry mechanism (14). In an experiment using a ZIKV-infected mouse model, sTIM1dMLDR801 reduced ZIKV levels in the serum and spleen but did not inhibit PS-independent viral entry, and therefore, it was incapable of completely inhibiting viral replication (14). Furthermore, although mutants of MFG-E8 and TIM-1 inhibited the PS-binding receptor TIM-1-mediated ZIKV infection, widely used PS-binding molecules such as annexin V and β2GP1, with or without bavituximab, did not (14). These inhibition results may be due to differences in the requirement for PS during viral entry or to the extent to which PS exposed on the viral envelope can be completely masked. The *in vivo* and *in vitro* results indicate that it would be optimal to not only mask PS effectively but also eliminate spike proteins from the viral surface, which are necessary for viral entry. We, therefore, focused on the capability of CyD derivatives to remove phospholipids and proteins from the membrane although this depends on the structure and composition of the membrane and the type and concentration of the CyD derivative. In particular, methyl-β-CyD and methyl-γ-CyD can remove PS from liposomes, and methyl-β-CyD can induce the release of substantial quantities of GPI-anchored Thy-1 and other transmembrane proteins including CD45 from T lymphoma cells (15, 16). These findings prompted us to investigate whether CyD derivatives can effectively mask the PS of the lipid envelope of SARS-CoV-2, thereby reducing the stability of the spike protein on the surfaces of viral particles, and ultimately the stability of the viral particles themselves.

In this study, the anti-SARS-CoV-2 activity of 19 available CyD derivatives was evaluated using the SARS-CoV-2 Wuhan, Beta, and Delta variants. The findings indicated that DMA-β-CyD exhibited the most pronounced antiviral efficacy against SARS-CoV-2. DMA-β-CyD interacts with aminophospholipids on the viral surface and reduces the stability of viral particles, inhibiting viral entry. In addition, DMA-β-CyD similarly demonstrated antiviral activity against JEV. These results suggest that DMA-β-CyD may represent a novel antiviral agent targeting the viral lipid envelope.

## MATERIALS AND METHODS

### Cell preparation

Vero cells and Vero E6/TMPRSS2 cells were obtained from the JCRB Cell Bank, while Vero E6 and A549 cells were sourced from the American Type Culture Collection. Vero cells and Vero E6 cells were maintained in EMEM (FUJIFILM Wako Pure Chemical Corporation) supplemented with 10% FBS (Capricorn Scientific GmbH). Vero E6/TMPRSS2 cells were maintained in DMEM (FUJIFILM Wako Pure Chemical Corporation) supplemented with 10% FBS and 1 mg/mL G-418 (Nacalai Tesque, Inc.). A549 cells were maintained in DMEM supplemented with 10% FBS, 100 U/mL penicillin G, 0.1 mg titer/mL streptomycin, and 62.5 µg/mL gentamicin. All cells were cultured at 37°C in 5% CO₂.

### Viral preparation

The SARS-CoV-2 Wuhan (hCoV-19/Japan/TY/WK-521/2019), Beta (hCoV-19/Japan/TY8-612/2021), and Delta (hCoV-19/Japan/TKY76137/2021) variants were cultivated in Vero E6/TMPRSS2 in a BSL3 laboratory. Vero E6/TMPRSS2 cells were seeded at a concentration of 3.0 × 10^6^ cells/mL in a 75 cm^2^ tissue culture flask and incubated for 24 h at 37°C in 5% CO_2_. Cells were infected with each SARS-CoV-2 variant at M.O.I. = 0.05 and incubated for 72 h. The supernatant of each culture was collected through DISMIC-25cs (0.45 µm, Toyo Roshi Kaisha, Ltd.) and stored at -20°C. Viral infectivity titers were determined by the TCID₅₀ method.

Additionally, the SARS-CoV-2 Wuhan variant was also propagated in A549 cells transiently expressing human ACE2. Briefly, A549 cells (4.8 × 10⁶ cells/dish) were transfected with 24 µg of a plasmid encoding human ACE2, constructed by inserting the ACE2 gene into the KpnI and XbaI sites of the pcDNA4/Myc-His A vector (Thermo Fisher Scientific Inc.), which was kindly provided by Dr. Kurogi (University of Miyazaki). Transfection was performed using 60 µL of PEI Max reagent (Polysciences, Inc.), following the manufacturer’s protocol. After ACE2 expression was confirmed, cells were infected with the Wuhan variant under the same conditions described above, and virus-containing supernatants were similarly collected and stored.

To prepare JEV, Vero cells were seeded at 2 × 10⁶ cells per flask in 15 mL of EMEM supplemented with 10% fetal bovine serum (FBS). Cells were infected with JEV at M.O.I. = 0.1 and incubated for 48 h. After incubation, culture supernatants were collected, and 10 g of PEG6000 was added per 100  mL of supernatant, along with NaCl to a final concentration of 0.5 M. The mixture was centrifuged at 11,291.8 × *g* for 30 min, and the resulting viral pellet was resuspended in 1  mL of TN buffer (10 mM Tris-HCl, pH 7.5, 100 mM NaCl). Following a second centrifugation at 11,291.8 × *g* for 30 min, the pellet was ultracentrifuged through a 10%–40% (wt/wt) sucrose density gradient at 259,000 × *g* for 90  min at 4°C. The virus-containing fraction was then buffer-exchanged using a PD-10 desalting column (Cytiva), and the final viral preparation was stored as the working stock. The titer was calculated by the TCID_50_ method.

### Antiviral efficacy test

To identify effective compounds among 19 CyD derivatives against the three SARS-CoV-2 variants, Vero E6/TMPRSS2 cells (5 × 10³ cells/well) were seeded in 96-well flat-bottom plates (AS ONE Corporation). After 24 h, the cells were infected with SARS-CoV-2 variants at M.O.I. = 0.025 in the presence of CyD derivative solutions and incubated for an additional 72 h at 37°C in 5% CO₂. For CyD derivatives that exhibited cytotoxicity at concentrations below 10 mM, the highest nontoxic concentration was used to prepare antiviral test solutions. For those without detectable cytotoxicity at 10 mM, this concentration was adopted as the maximum dose. All test solutions were serially diluted fourfold and used in the antiviral efficacy test. Wells in which no visible cytopathic effect was observed under a microscope were defined as negative for infection.

To determine the IC₅₀ values of 2,6-di-*O*-methyl-β-cyclodextrin (DM-β-CyD) and DMA-β-CyD, Vero E6/TMPRSS2 cells (5 × 10^3^ cells/well) were seeded on 96-well flat-bottom plates. After 24 h, the cells were exposed to the virus at M.O.I. = 0.025 in the presence of the compounds for 1 h, the virus was then washed out, and the cells were cultured in the presence or absence of the compounds. After 72 h, culture supernatants were collected, and viral RNA was extracted using the QIAamp Viral RNA Mini Kit (QIAGEN N.V.). Quantitative reverse transcription PCR (RT-qPCR) was then performed using the One Step PrimeScript III RT-qPCR Mix (Takara Bio) in accordance with the manufacturer’s protocol. The primers and probe targeting the SARS-CoV-2 nucleocapsid gene were as follows: forward primer, 5′-GACCCCAAAATCAGCGAAAT-3′; reverse primer, 5′-TCTGGTTACTGCCAGTTGAATCTG-3′; and probe, 5′-(6-FAM)-ACCCCGCATTACGTTTGGTGGACC-(BHQ1)-3′. All assays were performed in triplicate (*n* = 3).

To evaluate the antiviral efficacy of DMA-β-CyD against JEV, the IC₅₀ value was determined as follows. Vero cells (5 × 10^3^ cells/well) were seeded on 96-well flat-bottom plates. After 24 h, the cells were exposed to JEV (Beijing-1 variant) at M.O.I. = 0.025 in the presence of the compounds for 1 h; the virus was then washed out, and the cells were cultured in the presence or absence of the compounds. After 72 h, culture supernatants were collected, and viral RNA was extracted using the QIAamp Viral RNA Mini Kit. Quantitative reverse transcription PCR (RT-qPCR) was then performed using the One Step PrimeScript III RT-qPCR Mix and JEV-1 Primer/Probe Mix (Takara Bio) in accordance with the manufacturer’s protocol. All assays were performed in triplicate (*n* = 3).

### Cytotoxicity assay

A solution of each CyD derivative, prepared in DMEM supplemented with 10% FBS and 1 mg/mL G-418, was filtered and sterilized using a DISMIC-25cs 0.45 µm filter. Vero E6 cells and Vero E6/TMPRSS2 cells (5 × 10^3^ cells/well) were seeded on 96-well flat-bottom plates. After 24 h, the cells were treated with CyD derivatives and further incubated for 72 h in a humidified incubator at 37°C with 5% CO₂. Cytotoxicity was assessed under a microscope.

To assess the cytotoxicity of DM-β-CyD and DMA-β-CyD—both of which demonstrated antiviral activity in the initial screening—Vero E6/TMPRSS2 cells (5  ×  10³ cells/well) were seeded on 96-well flat-bottom plates. After 24 h, each compound was tested at concentrations up to 100 mM using the CCK-8 assay (DOJINDO Laboratories), following the manufacturer’s protocol.

To assess the cytotoxicity of E-64D, Vero E6 cells or Vero E6/TMPRSS2 cells (5  ×  10³ cells/well) were seeded on 96-well flat-bottom plates. After 24 h, E-64D dissolved in DMSO was tested at concentrations up to 320 µM using the CCK-8 assay. As a control, DMSO was added to all wells to maintain a final concentration of 0.0125%.

To further assess the cytotoxicity of DMA-β-CyD against Vero cells (5 × 10^3^ cells/well), the target cells for JEV, the compound was tested at concentrations up to 100  mM using the CCK-8 assay.

### Direct viral inactivation test

Vero E6/TMPRSS2 cells were seeded on a 96-well flat-bottom plate and incubated for 24 h at 37°C in 5% CO_2_. CyD derivatives were dissolved in DMEM supplemented with 10% FBS and 1 mg/mL G-418 to prepare working solutions at final concentrations of 5 mM for DM-β-CyD and 10 mM for DMA-β-CyD. The solutions were sterilized using a DISMIC-25cs 0.45 µm filter. Spin columns were prepared by packing Pierce Microcentrifuge Tubes (1.5 mL; Thermo Fisher Scientific Inc.) with Sepharose CL-4B (Global Life Sciences Technologies Japan K.K.). SARS-CoV-2 (Wuhan variant, 2.1 × 10^7^ TCID_50_/mL; Beta variant, 3.75 × 10^6^ TCID_50_/mL; Delta variant, 1.58 × 10^6^ TCID_50_/mL, 100 µL) was mixed with 100 µL of each CyD derivative solution and incubated for either 10 or 180 min. To effectively separate the virus from the CyD derivative, 200 µL of the treated sample was carefully layered onto spin columns pre-packed with 600 µL of Sepharose CL-4B slurry in 1.5 mL Pierce Microcentrifuge Tubes. The columns were then centrifuged at 50 × *g* for 1  min. The resulting flow-through fractions were subsequently inoculated onto Vero E6/TMPRSS2 cells, and viral infectivity titers were determined using the Behrens–Kärber method and expressed as TCID₅₀/mL.

To further investigate the mechanism of action of DMA-β-CyD, both Vero E6 cells and Vero E6/TMPRSS2 cells were used. The SARS-CoV-2 Wuhan variant (2.11 × 10^7^ TCID_50_/mL, 100 µL) was incubated with 20 mM DMA-β-CyD (100 µL) for 180 min, and then the residual DMA-β-CyD was removed by gel filtration using Sepharose CL-4B. Each cell type was seeded at 5 × 10³ cells/well in a 96-well flat-bottom plate. After 24 h, the cells were infected with the DMA-β-CyD-treated Wuhan variant and then incubated for an additional 72 h. Viral infectivity titers were then determined by the microscopic evaluation of CPE or by measuring absorbance using the CCK-8 assay. In addition, the infectivity titer of the DMA-β-CyD-treated Wuhan variant was also assessed in cells pretreated with E-64D (10 µM, 180 min; Peptide Institute, Inc.).

### ELISA

To determine whether DMA-β-CyD inhibits the binding of the SARS-CoV-2 spike protein to ACE2, an ACE2:SARS-CoV-2 Spike S1 Inhibitor Screening Assay Kit (BPS Bioscience, Inc.) was used. DMA-β-CyD was dissolved in PBS(-) and prepared at final concentrations of 0.1 mM and 10 mM. The assay was performed in accordance with the manufacturer’s instructions, and the absorbance was measured using a Synergy Neo2 microplate reader (BioTek Instruments, Inc.).

### Inhibition of coagulation activity determined by activated partial thromboplastin time measurement

The activities of both the intrinsic and extrinsic coagulation pathways were evaluated in recalcified human plasma activated upon contact with a negatively charged surface provided by a glass test tube. A purified SARS-CoV-2 viral suspension in Hank’s Balanced Salt Solution (HBSS, 50 µL; protein concentration = 1 mg/mL) was mixed with an equal volume of normal pooled human plasma (Alpha Laboratories, CCN-10) in a glass tube and incubated at 37°C for 1 min. Subsequently, 50 µL of 20 mM CaCl₂, prewarmed to the appropriate temperature, was added to the mixture, which was then incubated at 37°C. Clotting time was determined by visual inspection using a stopwatch and was defined as the interval until a visible fibrin clot appeared. The coagulation-modulating activity of live viral particles treated with 10 mM DMA-β-CyD or 5 mM DM-β-CyD was assessed after various incubation periods. To investigate whether the inclusion complex-forming ability of DMA-β-CyD was required for its effect, SARS-CoV-2 was pretreated for 80 min with 10 mM DMA-β-CyD complexed with an equimolar amount of 1-adamantanecarboxylic acid (Tokyo Chemical Industry Co., Ltd.), as described by Cid-Samamed et al. (17), and changes in clotting time were measured. As a negative control, 50 µL of HBSS was added to plasma instead of the virus.

To evaluate the coagulation-modulating activity of JEV, the same activated partial thromboplastin time assay protocol as described above for SARS-CoV-2 was applied. Briefly, JEV (50 µL; protein concentration = 1 mg/mL) was incubated at 28 or 37°C for various incubation periods with either 10  mM DMA-β-CyD or 5 mM DM-β-CyD. After incubation, 50 µL of pooled human plasma was added, followed by 50 µL of prewarmed 20 mM CaCl₂. Clotting time was recorded as the time to visible fibrin clot formation.

### Entry assay

Vero E6/TMPRSS2 cells (5 × 10^3^ cells/well) were seeded on a 96-well flat-bottom plate (AS ONE Corporation) and incubated for 24 h at 37°C in 5% CO₂. The SARS-CoV-2 Wuhan variant, derived from Vero E6/TMPRSS2 cells or from A549 cells transiently expressing human ACE2, was preincubated with DMA-β-CyD in a test tube at final concentrations of 1 mM and 10 mM for 180 min at 37°C. Following preincubation, the mixture was subjected to gel filtration using spin columns packed with 360 µL of Sepharose CL-4B slurry in Pierce Microcentrifuge Tubes (1.5  mL), into which 120 µL of a virus-containing sample was carefully loaded to effectively separate the virus from DMA-β-CyD. The resulting virus-containing flow-through fraction was added to the cells at M.O.I. = 0.025, and the plates were incubated for 4 h at 37°C in 5% CO₂. After this initial 4 h exposure, the supernatant was completely removed from each well, and the cells were gently washed three times with PBS(-). The cells were then detached using 0.25% trypsin-0.02% EDTA solution and subsequently washed once more with PBS(-) to remove residual enzymes and media. Total RNA was then extracted from the harvested cells using an RNeasy Mini Kit (QIAGEN N.V.) according to the manufacturer’s protocol. Quantitative RT-PCR was performed using the SARS-CoV-2 RT-qPCR Detection Kit (FUJIFILM Wako Pure Chemical Industries, Ltd.) following the manufacturer’s protocol. Amplification was conducted on a LightCycler 96 system (F. Hoffmann-La Roche Ltd.). As a positive control for entry inhibition, the SARS-CoV-2 Wuhan variant was preincubated with heat-inactivated convalescent serum derived from a COVID-19 patient (a kind gift from Dr. Matsushita, Kumamoto University) at dilutions of 1:20 or 1:200 for 180 min at 37°C. The virus–serum mixture was then subjected to the same entry assay procedure described above.

### Western blot analysis

To determine whether DMA-β-CyD induces physical disruption of viral particles, the SARS-CoV-2 Wuhan variant, derived from Vero E6/TMPRSS2 cells, was preincubated with 10 mM DMA-β-CyD in a test tube for 180 min at 37°C. A viral solution (2.11 × 10^7^ TCID_50_/mL, 50 µL) was used for this assay. Following preincubation, viral particles were collected by ultracentrifugation at 100,000 × *g* for 1 h at 4°C. The resulting viral pellet was washed once with HBSS to remove unbound compounds or debris. After washing, the pellet was resuspended and lysed in incubation buffer (125 mM Tris-HCl, 4% SDS, 20% glycerol, pH 6.8) containing a protease inhibitor cocktail (Nacalai Tesque, Inc.). For comparison, the virus was also treated with heat-inactivated convalescent serum derived from a SARS-CoV-2-infected patient (a kind gift from Dr. Matsushita, Kumamoto University), diluted at 1:20 in DMEM, under the same conditions (180 min at 37°C). The virus was then subjected to identical washing and lysis steps. The lysates were subjected to SDS–PAGE, followed by western blotting using the same convalescent serum, which contained antibodies against the N protein and spike protein.

To confirm ACE2 expression in transfected cells, A549 cells (4.8 × 10⁶ cells/dish) were transfected with 24 µg of a human ACE2 expression plasmid, constructed by inserting the human ACE2 gene. Transfection was performed using 60 µL of PEI Max reagent (Polysciences, Inc.) according to the manufacturer’s instructions. After 48 h, cells were washed with PBS(-), detached using 0.25% trypsin-0.02% EDTA solution, and collected by centrifugation. The cell pellet was lysed with RIPA buffer, and lysates were subjected to SDS–PAGE followed by western blotting using a rabbit anti-ACE2 monoclonal antibody (Abcam plc).

### Structural stability assay of SARS-CoV-2 virions

To evaluate the sizes of particles obtained by gel filtration, the SARS-CoV-2 Wuhan variant (2.11 × 10^7^ TCID_50_/mL, 120 µL) was incubated with 2 µg of the mouse monoclonal antibody against the S2 protein (GeneTex, Inc.) for 180 min at 37°C. The mixture was then subjected to gel filtration using spin columns packed with 360 µL of Sepharose CL-4B slurry in Pierce Microcentrifuge Tubes (1.5 mL), into which 120 µL of a virus-containing sample was carefully loaded. As molecular size markers, 2 µg of mouse IgG (~150 kDa) or IgM (~900  kDa) was included to determine the approximate size of proteins eluted in the flow-through fraction obtained after centrifugation. The resulting samples were analyzed by western blotting using peroxidase-conjugated AffiniPure goat anti-mouse IgG (Jackson ImmunoResearch Inc.). This antibody is known to react with the light chains of mouse IgM and with immunoglobulins of other species.

Next, the SARS-CoV-2 Wuhan variant (2.11 × 10^7^ TCID_50_/mL, 60 µL) was incubated for 180 min at 37°C with either 60 µL of 2  mM or 20 mM DMA-β-CyD, or with heat-inactivated convalescent serum (diluted 1:20 or 1:200) derived from a SARS-CoV-2-infected patient. These viral preparations were then subjected to gel filtration as described above. The flow-through fractions (100 µL), containing the virus, were added to microplates precoated with anti-Receptor Binding Domain (RBD) antibodies (Hakarel Inc.) and incubated for 120 min at room temperature. After three washes with the washing buffer provided in the kit, the quantity of the virus bound via capture by the anti-RBD antibody was determined by RT-PCR, as described in the Antiviral Efficacy Test subsection of Materials and Methods.

## RESULTS

### DMA-β-CyD possesses antiviral activity against SARS-CoV-2 variants

**Fig 1:**
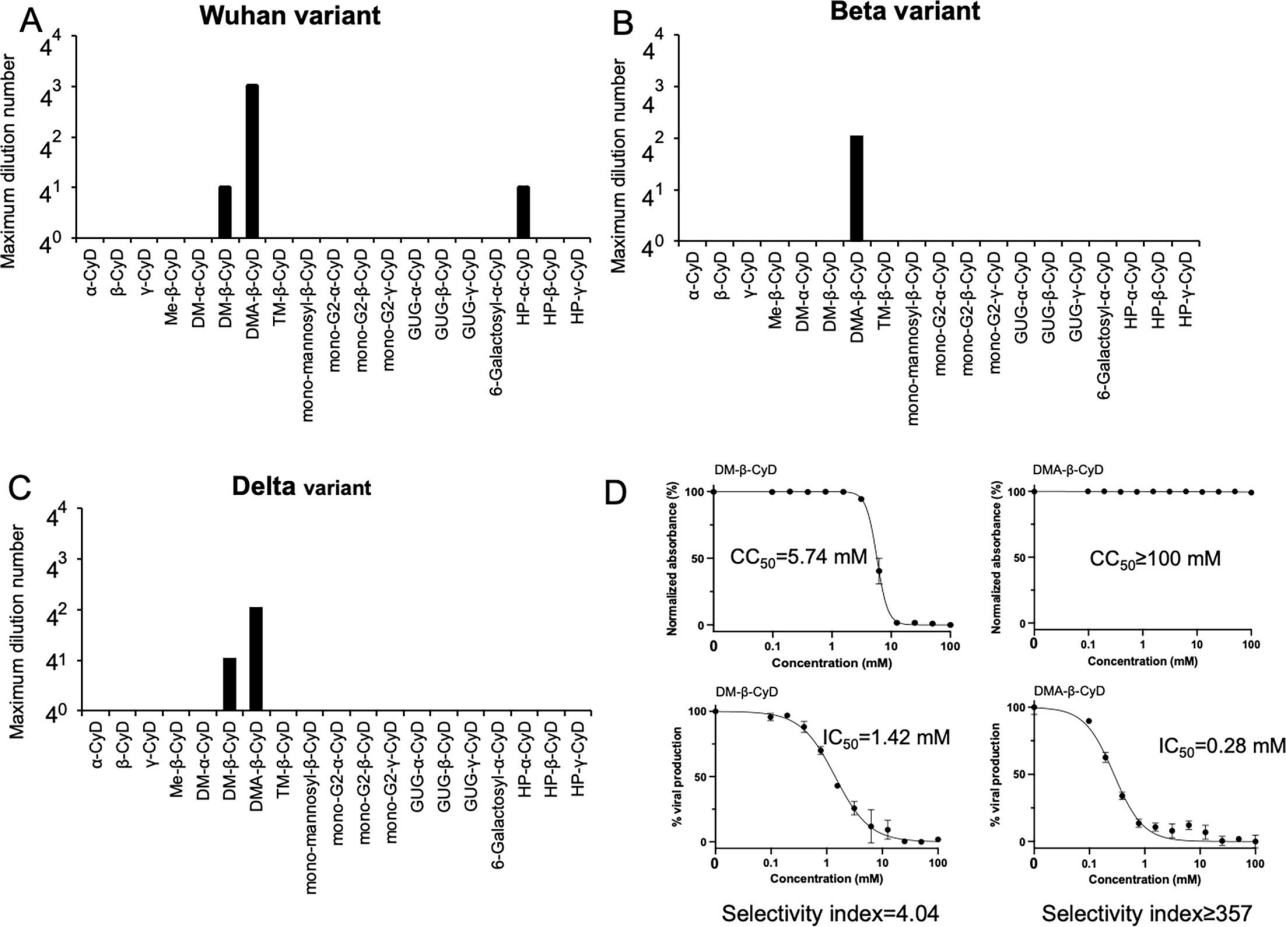
Evaluation of the anti-SARS-CoV-2 activity of CyD derivatives. Inhibitory effects on (**A**) Wuhan, (**B**) Beta, and (**C**) Delta variants. Each compound was serially diluted fourfold for the assessment of antiviral activity, and the maximum dilution showed the complete absence of the cytopathic effect. (**D**) Cytotoxicity and antiviral activity of DM-β-CyD and DMA-β-CyD against SARS-CoV-2. VeroE6/TMPRSS2 cells were exposed to the SARS-CoV-2 Wuhan variant. Normalized absorbance (%) values were calculated by setting the absorbance at 450 nm of untreated, non-cytotoxic control cells to 100%. A decrease in absorbance reflects reduced cell viability owing to cytotoxicity. The percent viral production in the VeroE6/TMPRSS2 cell culture supernatant was determined by RT-qPCR analysis. Each compound was tested at least three times. The data are shown as means  ±  standard deviations (SD).

The 19 CyD derivatives used in this study to analyze the antiviral effects against SARS-CoV-2 are listed in Table 1. From the results of the analysis of host-guest interactions, CyD derivatives have been found to form complexes with natural hydrophobic molecules that constitute lipid bilayer membranes. Specifically, α-CyD forms complexes with phospholipids (18, 19), while β-CyD forms complexes with membrane cholesterol as a cellular target (20). Furthermore, methyl-β-CyD and methyl-γ-CyD have been demonstrated to be effective in the extraction of PS from liposomes, and methyl-β-CyD has been reported to preferentially extract cholesterol and selectively extract transmembrane proteins such as CD45 from T lymphocytes (15, 16). However, it has also been found that the addition of diverse types and numbers of functional groups to the hydroxyl groups at the 2-, 3-, and 6-positions of glucose significantly influences these effects. The toxicity of the 19 CyD derivatives used in this study to VeroE6/TMPRSS2 cells was evaluated in advance using a microscope, and the highest concentrations that showed no toxicity are shown in the rightmost column in Table 1. In Fig. 1A, to assess the anti-SARS-CoV-2 activity of each CyD derivative, compounds that exhibited cytotoxicity at concentrations of 10  mM or lower were evaluated at their highest non-toxic concentration. Conversely, derivatives that did not exhibit any cytotoxicity at 10 mM were tested at 10 mM as the maximum concentration. From these maximum concentrations, a series of fourfold serial dilutions were prepared to determine the dilution at which complete inhibition of the cytopathic effect induced by SARS-CoV-2 infection was observed (Fig. 1). In an infection experiment against the Wuhan variant, DM-β-CyD, DMA-β-CyD, and hydroxypropyl-α-cyclodextrin (HP-α-CyD) showed infection inhibitory activity (Fig. 1A), with DMA-β-CyD completely inhibiting the cytopathic effect at the highest dilution of 64-fold (156 µM). Similarly, when evaluated against the β variant, only DMA-β-CyD completely inhibited the cytopathic effect at the highest dilution of 16-fold (625 µM) (Fig. 1B). Against the Delta variant, DM-β-CyD and DMA-β-CyD showed infection inhibitory activity, with DMA-β-CyD completely inhibiting the cytopathic effect at the highest dilution of 64 times (156 µM) (Fig. 1C). Bolland et al. (21), Bezerra et al. (22), and Raïch-Regué et al. (23) demonstrated that β-CyD derivatives such as methyl-β-CyD and HP-β-CyD inhibited SARS-CoV-2 spike-protein-mediated entry and cell fusion by depleting cholesterol from the host cell membrane. It has been reported that the activity in removing cholesterol is in the following order: DM-β-CyD > methyl-β-CyD > hydroxypropyl-β-CyD (24). Surprisingly, DMA-β-CyD—a compound that releases cholesterol less effectively from lipid bilayers than DM-β-CyD (25)(25—exhibited greater potency in inhibiting SARS-CoV-2 spike-protein-mediated entry and fusion than these CyD derivatives. These results suggest that DMA-β-CyD may possess antiviral activity against SARS-CoV-2 variants, which does not involve cholesterol release.

To assess the potency of DMA-β-CyD and DM-β-CyD in more detail, we calculated the half-maximal inhibitory concentration (IC₅₀), the 50% cytotoxic concentration (CC₅₀), and the selectivity index (SI = CC₅₀/IC₅₀), as shown in Fig. 1D. The IC₅₀ values for DMA-β-CyD and DM-β-CyD against SARS-CoV-2 infection were 0.28 and 1.42 mM, and the CC₅₀ values were ≥100 mM and 5.74 mM, respectively. The SI values of DMA-β-CyD and DM-β-CyD were ≥357 and 4.04, respectively. The relatively high SI value of DMA-β-CyD (≥357) suggests that it may serve as a promising lead compound with a potentially distinct mechanism of antiviral action. To further elucidate this mechanism, we next examined whether its antiviral effects involved a direct impact on the structural integrity of SARS-CoV-2 virions.

**TABLE 1:** List of cyclodextrin derivatives studied in this work^*a*^

Compound	*n* ^*b*^	R1	R2	R3	D.S.^*c*^	M.W.^*d*^	Highest non-toxic concentration [mM]^*e*^
α-CyD	6	-H	-H	-H	–^*f*^	972	5.00
β-CyD	7	-H	-H	-H	–	1,135	≥10.0
γ-CyD	8	-H	-H	-H	–	1,297	2.50
Me-β-CyD	7		-H or -CH3		12.2	1,303	2.50
DM-α-CyD	6	-CH3	-H	-CH3	12	1,141	2.50
DM-β-CyD	7	-CH3	-H	-CH3	14	1,331	5.00
DMA-β-CyD	7	-CH3	-H or -COCH3	-CH3	7	1,624	≥100.0
TM-β-CyD	7	-CH3	-CH3	-CH3	21	1,430	1.25
Mono-mannosyl-β-CyD	7	-H	-H	-H or -mannose	1	1,299	5.00
Mono G2-α-CyD	6	-H	-H	-H or -maltose	1	1,297	≥10.0
Mono G2-β-CyD	7	-H	-H	-H or -maltose	1	1,459	≥10.0
Mono G2-γ-CyD	8	-H	-H	-H or -maltose	1	1,621	5.00
GUG-α-CyD	6	-H	-H	-H or -GUG	1	1,311	5.00
GUG-β-CyD	7	-H	-H	-H or -GUG	1	1,473	10.0
GUG-γ-CyD	8	-H	-H	-H or -GUG	1	1,635	5.00
6-Galactosyl-α-CyD	6	-H	-H	-H or -galactose	1	1,135	≥10.0
HP-α-CyD	6		-H or -CH2OH(CH2)OH		4.1	1,275	≥10.0
HP-β-CyD	7		-H or -CH2OH(CH2)OH		4.8	1,471	≥10.0
HP-γ-CyD	8		-H or -CH2OH(CH2)OH		4.2	1,616	5.00

^*a*^ Me, randomly-methylated; DM, 2,6-di-O -methyl; TM, 2,3,6-tri-O -methyl; DMA, 2,6-di-O -methyl-3-O -acetyl-; HP, hydroxypropyl; G2, maltosyl; GUG, Glucuronyl-glucosyl.

^*b
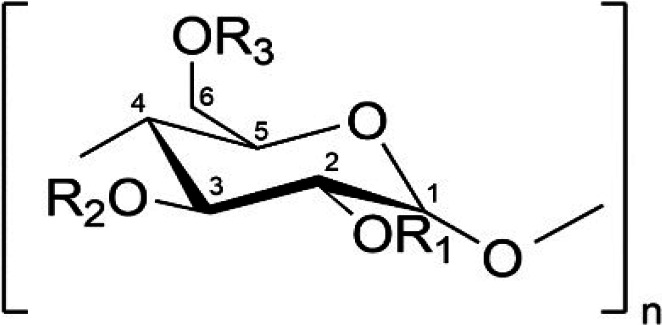
*^ .

^*c*^ Average degree of substitution.

^*d*^ Molecular weight.

^*e*^ The test substance was prepared in twofold dilutions with 10 mM as the highest concentration.The values represent the highest concentration that is not toxic to Vero E6/TMPRSS2 cells.

^*f*^ –, not applicable.

### DMA-β-CyD exerts a direct effect on the virus, leading to a reduction in infectious viral titer

**Fig 2:**
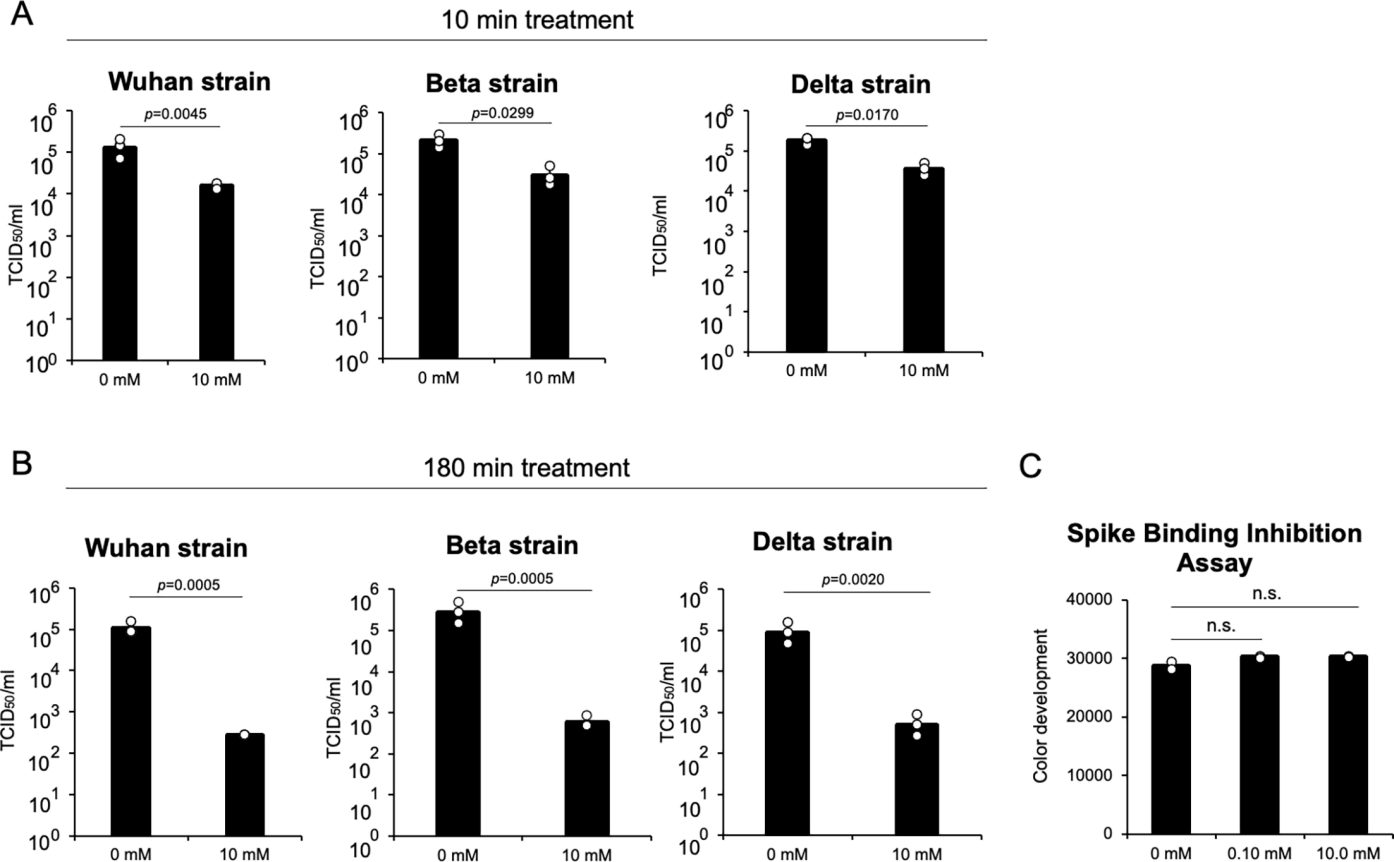
DMA-β-CyD acts directly on SARS-CoV-2 and reduces its infectivity titer. DMA-β-CyD was directly exposed to different SARS-CoV-2 variants for (**A**) 10 min and (**B**) 180 min. Viral infectivity titers were expressed in TCID_50_/mL, and significant differences (Student’s *t*-test) are indicated in the figures. Mean values from at least three independent experiments are shown, and error bars indicate SD. (**C**) Competitive inhibition experiment with DMA-β-CyD. Recombinant ACE2 was used, and DMA-β-CyD was pre-incubated before the loading of the recombinant spike protein. Statistical significance was calculated using one-way analysis of variance (ANOVA) followed by Dunnett’s test. n.s. indicates not significant. The mean values of at least three independent experiments are shown. The error bars denote SD.

To better understand the mechanism of action of DMA-β-CyD against SARS-CoV-2, the compound was directly applied to the virus to evaluate its impact on viral infectivity. Three distinct SARS-CoV-2 variants were treated with 10 mM DMA-β-CyD for either 10 or 180 min, followed by gel filtration to remove the compound. Infectivity was then assessed using VeroE6/TMPRSS2 cells. After a 10 min exposure, viral titers were significantly reduced: the Wuhan variant demonstrated a decrease from 1.29 × 10⁵ to 1.62 × 10⁴ TCID₅₀/mL; the Beta variant, from 2.04 × 10⁵ to 2.88 × 10⁴ TCID₅₀/mL; and the Delta variant, from 1.82 × 10⁵ to 3.63 × 10⁴ TCID₅₀/mL (Fig. 2A). Extending the treatment to 180 min led to even greater reductions in viral titers: the Wuhan variant dropped from 1.05 × 10⁵ to 2.75 × 10² TCID₅₀/mL; the Beta variant, from 2.75 × 10⁵ to 5.89 × 10² TCID₅₀/mL; and the Delta variant, from 8.71 × 10⁴ to 4.90 × 10² TCID₅₀/mL, demonstrating a substantial decline in infectivity across all variants (Fig. 2B). Saud et al. (3) used lipidomics to show that the viral envelope of SARS-CoV-2 is mainly composed of phospholipids and contains almost no cholesterol or sphingolipids, indicating a significant difference from the host membrane. These findings suggest that DMA-CyD may act on lipids other than cholesterol or sphingolipids in the viral lipid envelope or on proteins on the viral surface to reduce viral infectivity.

To determine whether the direct effect of DMA-β-CyD on viral particles was related to its inhibition of the spike protein binding to ACE2, we performed a spike protein-ACE2-binding assay. The results showed that DMA-β-CyD did not interfere with this interaction, suggesting that its antiviral effect may involve a direct disruption of the viral lipid envelope itself rather than the inhibition of spike protein-ACE2 binding (Fig. 2C).

### DMA-β-CyD inhibits plasma coagulation mediated by SARS-CoV-2

**Fig 3:**
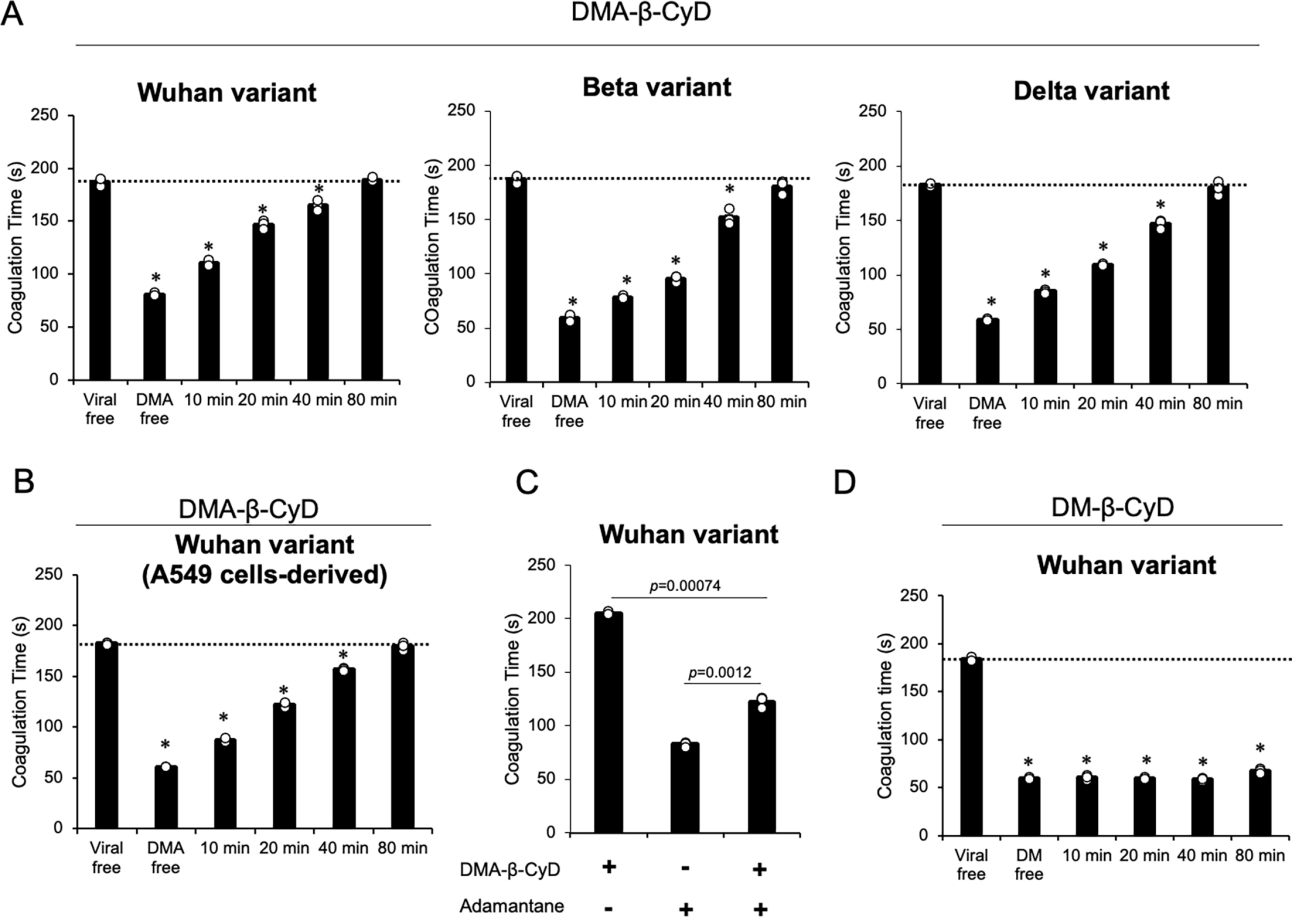
DMA-β-CyD prolongs the virus-induced reduction in clotting time in human serum by masking aminophospholipids in the viral lipid envelope. (**A**) The Wuhan, Beta, and Delta variants prepared in Vero E6/TMPRSS2 cells were treated with DMA-β-CyD for the indicated times, followed by a clotting assay. (**B**) The Wuhan variant, prepared in A549 cells overexpressing ACE2, was treated with DMA-β-CyD for the indicated times, followed by a clotting assay. (**C**) To examine the significance of the molecular encapsulation activity of DMA-β-CyD, pretreatment with 10 mM DMAβCyD with equimolar (10 mM) 1adamantanecarboxylic acid was performed to assess its effect on the virus-induced reduction in clotting time. (**D**) The Wuhan variant, prepared in Vero E6/TMPRSS2 cells, was treated with DM-β-CyD for the indicated times, followed by a clotting assay. *P* values were determined using one-way ANOVA (Dunnett’s test). * indicates a statistically significant difference in clotting time between groups untreated and treated with DMA-β-CyD or DM-β-CyD (*P* < 0.05). Data represent the means of at least three independent experiments, and error bars indicate SD.

We focused on the effect of DMA-β-CyD on the viral lipid envelope. While DMA-β-CyD did not induce hemolysis even at 100 mM, the concentrations at which β-CyD and DM-β-CyD caused 50% hemolysis were 4 mM and 1 mM, respectively (25). It has been revealed that one of the causes of CyD-induced hemolysis is the extraction of cholesterol and phospholipids from red blood cells through the formation of inclusion complexes (26). Furthermore, Saud et al. reported that the SARS-CoV-2 envelope is primarily composed of phospholipids, with minimal cholesterol and sphingolipids, making it structurally distinct from the host plasma membrane (3). From these findings, we hypothesized that DMA-β-CyD may preferentially interact with the lipid components unique to the viral envelope, rather than with those of normal cellular membranes. Considering that aminophospholipids, such as PS and phosphatidylethanolamine, are abnormally exposed on the outer leaflet of the SARS-CoV-2 envelope and accelerate plasma coagulation, we performed a clotting time assay to assess the modulatory effects of DMA-β-CyD. Treatment with 10 mM DMA-β-CyD markedly prolonged clotting time, reaching levels comparable to virus-free plasma after 80 min of incubation. This effect was observed across the Wuhan, Beta, and Delta variants (Fig. 3A). We further validated this observation using the Wuhan variant propagated in A549 cells. As shown in Fig. 3B, similar prolongation of clotting time was observed following treatment with 10 mM DMA-β-CyD, again reaching virus-free plasma levels after 80 min. These findings are consistent with those of Saud et al. (3), who reported that the proportions of aminophospholipids on the virion surface derived from A549 and Vero cells were comparable, approximately 48% and 52%, respectively. To assess the role of inclusion complex formation in this effect, the Wuhan variant was treated with DMA-β-CyD preincubated with 1-adamantanecarboxylic acid, an agent known to inhibit guest molecule binding. As shown in Fig. 3C, insufficient recovery of clotting time was observed, indicating that the inclusion capability of DMA-β-CyD is essential for masking the aminophospholipids in the lipid envelope of SARS-CoV-2. In contrast, treatment with 5 mM DM-β-CyD showed no effect on clotting time (Fig. 3D). These results suggest that the presence of a 3-acetyl group, in addition to the shared 2,6-di-*O*-methyl modifications, may significantly influence the physicochemical and biological properties of β-CyD derivatives that interact with aminophospholipids in the viral envelope of SARS-CoV-2.

### DMA-β-CyD inhibits SARS-CoV-2 infection of target cells through two distinct TMPRSS2-dependent and -independent pathways

**Fig 4:**
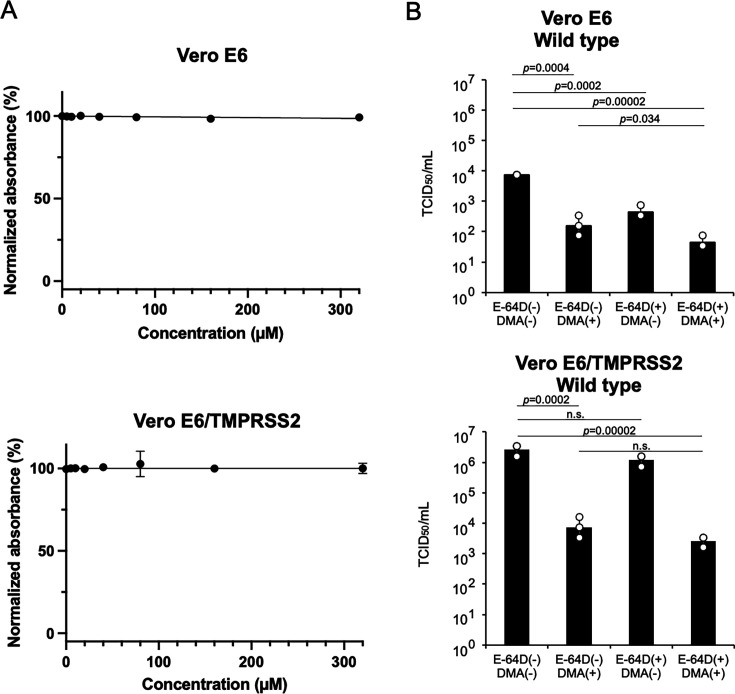
DMA-β-CyD exhibits antiviral activity through mechanisms other than the inhibition of phosphatidylserine-dependent viral entry. (**A**) E-64D cytotoxicity assay was performed against Vero E6 cells or Vero E6/TMPESS2 cells using CCK-8 assay. Normalized absorbance (%) values were calculated by setting the absorbance at 450 nm of untreated, non-cytotoxic control cells to 100%. The assay was conducted on at least three independent occasions. The data are shown as means ± SD. (**B**) The infectivity of SARS-CoV-2 treated with DMA-β-CyD was assessed using Vero E6 cells or Vero E6/TMPRESS2 cells with or without cotreatment with the cysteine protease inhibitor E-64D (10 µM). *P* values were determined using one-way ANOVA with Dunnett’s test. Data represent the means of at least three independent experiments, and the error bars denote SD. n.s. indicates not significant.

To prevent the spread of SARS-CoV-2, it is imperative to target the early stages of infection. In this study, we focused on investigating the inhibitory effect of DMA-β-CyD on two different routes of infection. SARS-CoV-2 binds to the ACE2 receptor and fuses at the cell membrane via the activation of the spike protein by the transmembrane serine protease TMPRSS2, or the virus is internalized by endocytosis and fuses at the endosomal membrane following activation of the spike protein by endosomal cathepsins. In addition, Bohan et al. reported that phosphatidylserine receptors enhance SARS-CoV-2 binding to cells and mediate internalization into endosomes (5). Notably, Koch et al. reported that in the presence of TMPRSS2, SARS-CoV-2 preferentially enters cells from the plasma membrane rapidly within 10 min, but when target cells lack TMPRSS2 expression, the virus is endocytosed and enters the cytoplasm via the activation of cathepsin L protease (27). Therefore, we first examined how DMA-β-CyD-treated SARS-CoV-2 infectivity was affected with or without cotreatment with the cysteine protease inhibitor E-64D using Vero E6 cells, which lack TMPRSS2 expression (28) and express ACE2 and the PS receptors AXL and TIM-1 (5). As shown in Fig. 4A, the cytotoxicity of E-64D against Vero E6 cells was examined, and E-64D was found to be nontoxic up to a concentration of 320 µM. Based on the recent report by Delphine et al. (29), in which Fig. S5 shows that E-64D at 10 µM effectively inhibited viral entry in VeroE6 cells but failed to do so in VeroE6/TMPRSS2 cells, we set the treatment concentration of E-64D to 10 µM and evaluated its effect together with DMA-β-CyD. As shown in Fig. 4B upper panel, the infectivity of the virus treated with 10 mM DMA-β-CyD for 180 min decreased from 7.34 × 10^3^ to 1.58 × 10^2^ TCID_50_/mL. This suggests that DMA-β-CyD masks the PS on the viral surface, inhibiting the interaction between PS and the PS receptor, and thus attenuating ACE2-dependent infection via endocytosis. In addition, when Vero E6 cells pretreated with E-64D were infected with a virus treated with 10 mM DMA-β-CyD, the infectivity decreased to 4.40 × 10 TCID_50_/mL, corresponding to a 2.22-log₁₀ reduction in TCID₅₀/mL compared with the control. On the other hand, we further investigated how the infectivity of DMA-β-CyD-treated SARS-CoV-2 changed with or without cotreatment with E-64D using Vero E6/TMPRSS2 cells, which highly express TMPRSS2. From the research results reported by Koch et al. (27), in Vero E6/TMPRSS2 cells that highly express TMPRSS2, the virus does not utilize endolysosomes but will mainly infect directly through the plasma membrane. Furthermore, Delphine et al. (29) reported that in Vero E6/TMPRSS2 cells, expressing TMPRSS2, E-64D was unable to sufficiently inhibit viral entry. As shown in Fig. 4B lower panel, TMPRSS2 expression increased the viral infectious titer to 2.64 × 10^6^ TCID_50_/mL, suggesting that TMPRSS2 expression increased the amount of SARS-CoV-2 that directly invades host cells through the plasma membrane. Furthermore, as expected, treatment with 10 µM E-64D did not significantly reduce virus infectivity. On the other hand, the infectivity of the virus treated with 10 mM DMA-β-CyD for 180 min decreased from 2.64 × 10^6^ to 7.34 × 10^3^ TCID_50_/mL. These results suggest that mechanisms other than the inhibition of phosphatidylserine-dependent viral entry may be involved in the antiviral activity of DMA-β-CyD.

### DMA-β-CyD inhibits viral entry by reducing virus particle stability

**Fig 5:**
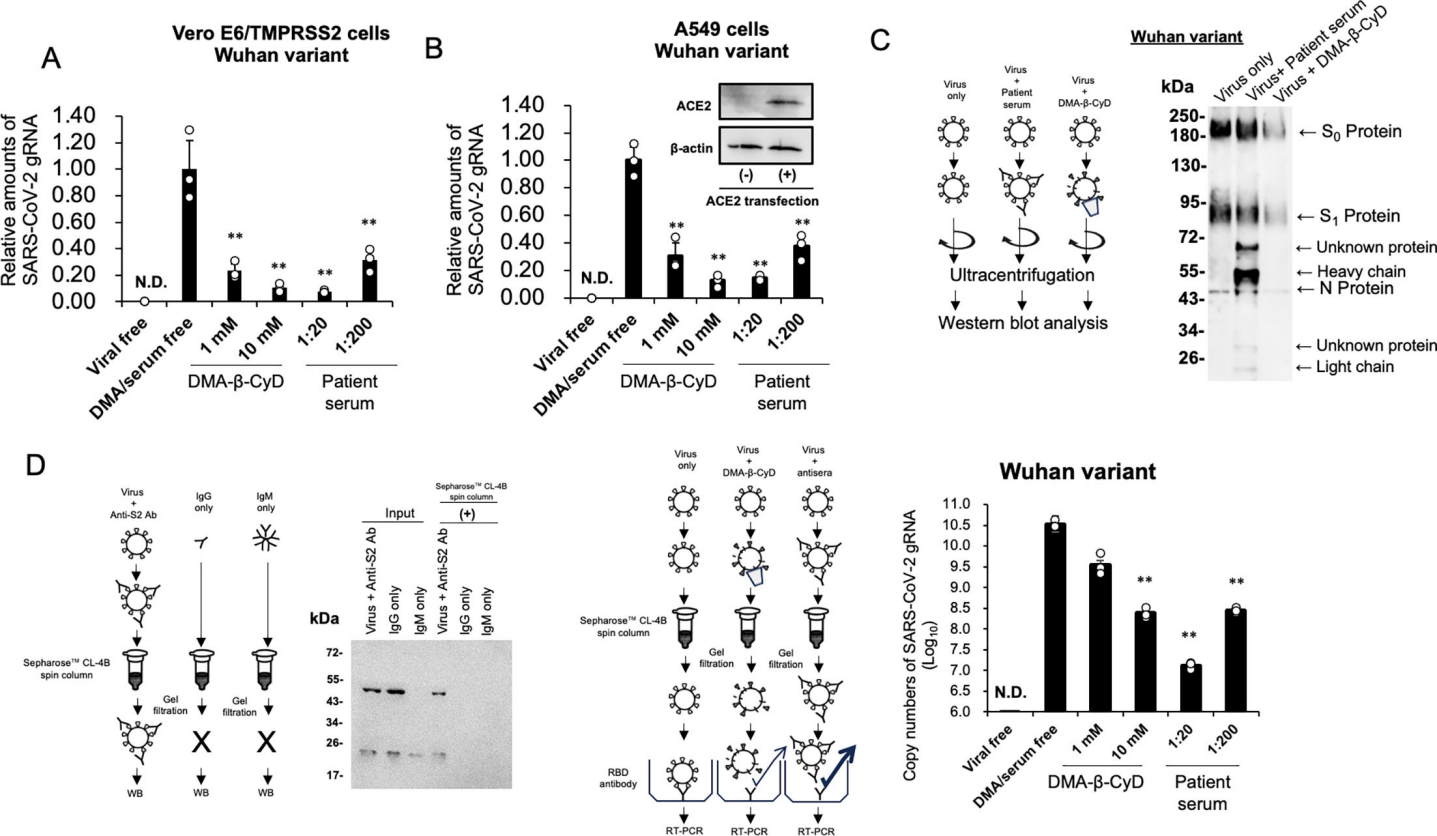
Inhibition of viral entry by DMA-β-CyD and evaluation of viral particle stability. (**A**) DMA-β-CyD was preincubated with the SARS-CoV-2 Wuhan variant, which was prepared in Vero E6/TMPRSS2 cells. Free DMA-β-CyD was removed by gel filtration, and the resulting viral particles were then used to infect cells. Four hours after infection, total RNA was extracted from Vero E6/TMPRSS2 cells, and gRNA was detected by RT-PCR. *P* values were determined by one-way ANOVA using Dunnett’s test (**P < 0.01). N.D. indicates that the SARS-CoV-2 phenotypic RNA (gRNA) was not detected. Data represent the means of at least three independent experiments, and error bars indicate SD. (**B**) DMA-β-CyD was preincubated with the SARS-CoV-2 Wuhan variant, which was prepared in A549 cells overexpressing ACE2. The efficiency of viral entry was similarly examined. Western blot analysis results, shown in the bar graph inset, confirm ACE2 expression in A549 cells. (**C**) The SARS-CoV-2 Wuhan variant, prepared in Vero E6/TMPRSS2 cells, was treated with anti-SARS-CoV-2 patient serum or DMA-β-CyD for 180 min, followed by virus purification by ultracentrifugation and western blot analysis using patient antiserum. (**D**) Evaluation of virus particle stability. (Left panel) The SARS-CoV-2 Wuhan variant, prepared in Vero E6/TMPRSS2 cells, was treated with an anti-S2 antibody and then subjected to gel filtration. Gel filtration was also performed using mouse IgG and IgM as molecular weight markers. The resulting flow-through fraction was analyzed by western blot analysis. (Right panel) The SARS-CoV-2 Wuhan variant, prepared in Vero E6/TMPRSS2 cells, was treated with DMA-β-CyD or anti-SARS-CoV-2 serum. The virus was purified by gel filtration, captured on a plate coated with anti-RBD antibodies, and the number of plate-bound viral particles was quantified by RT-qPCR. *P* values were determined by one-way ANOVA using Dunnett’s test (**P < 0.01). N.D. indicates that no detectable SARS-CoV-2 gRNA was found. Data represent the means of at least three independent experiments, and error bars indicate SD.

To clarify the antiviral effect of DMA-β-CyD other than the masking of PS on the viral surface, we performed a viral entry assay. Viral particles prepared in VeroE6/TMPRSS2 cells were treated *in vitro* with 1 or 10 mM DMA-β-CyD for 180 min. After separating the virus from DMA-β-CyD by gel filtration, the viral particles were used to infect VeroE6/TMPRSS2 cells. Four hours after infection, intracellular SARS-CoV-2 genomic RNA (gRNA) was detected, and its level decreased in a DMA-β-CyD-concentration-dependent manner, indicating that DMA-β-CyD suppressed SARS-CoV-2 entry (Fig. 5A). Similarly, viral particles derived from A549 cells transiently expressing ACE2 were treated *in vitro* with 1 or 10 mM DMA-β-CyD for 180 min, and entry assays demonstrated that the compound also inhibited the entry of these particles, regardless of the producer cell type (Fig. 5B). Furthermore, viral particles incubated for 180 min with the heat-inactivated convalescent serum obtained from a COVID-19 patient also showed diminished entry activity with increasing serum concentrations (Fig. 5A and B).

To compare the effects of DMA-β-CyD and the convalescent serum obtained from a COVID-19 patient, viral particles prepared in VeroE6/TMPRSS2 cells were treated under the same conditions and recovered by ultracentrifugation. Western blot analysis of the N protein and spike protein revealed that, while treatment with convalescent serum did not affect the band intensities of the two proteins, DMA-β-CyD treatment resulted in a marked reduction in the band intensities of both proteins (Fig. 5C). These findings suggest that DMA-β-CyD not only inhibits the interaction between viral aminophospholipids and PS receptors but also compromises the physical stability of viral particles, thereby reducing infectivity.

To corroborate the reduction in viral protein levels with direct evidence of particle destabilization, we conducted gel filtration and viral capture assays. As shown in Fig. 5D (left), SARS-CoV-2 Wuhan variant was incubated with the anti-S2 antibody for 180 min and then subjected to gel filtration using Sepharose CL-4B as described in Materials and Methods. The virus-antibody complex was eluted in the flow-through fraction, but IgG (approximately 150 kDa) and IgM (approximately 900 kDa) were not, suggesting that the viral particles were labeled by the anti-S2 antibody. Next, we similarly analyzed viral particles treated with DMA-β-CyD or the heat-inactivated convalescent serum. The resulting flow-through fraction was applied to plates coated with an antibody against the RBD of the spike protein. RNA quantification of the plate-bound viral particles revealed a concentration-dependent decrease in binding following treatment with the convalescent serum or DMA-β-CyD (Fig. 5D, right). The spike monomers and trimers released from viral particles treated with DMA-β-CyD are presumed to have molecular weights smaller than that of IgM and are, thus, unlikely to be retained in the flow-through fraction under these filtration conditions. Consequently, they would not interfere with the binding of intact viral particles to the anti-RBD antibody-coated plate. These results suggest that DMA-β-CyD impairs the structural integrity of viral particles, thereby reducing viral infectivity.

### DMA-β-CyD inhibits the infection of JEV

**Fig 6:**
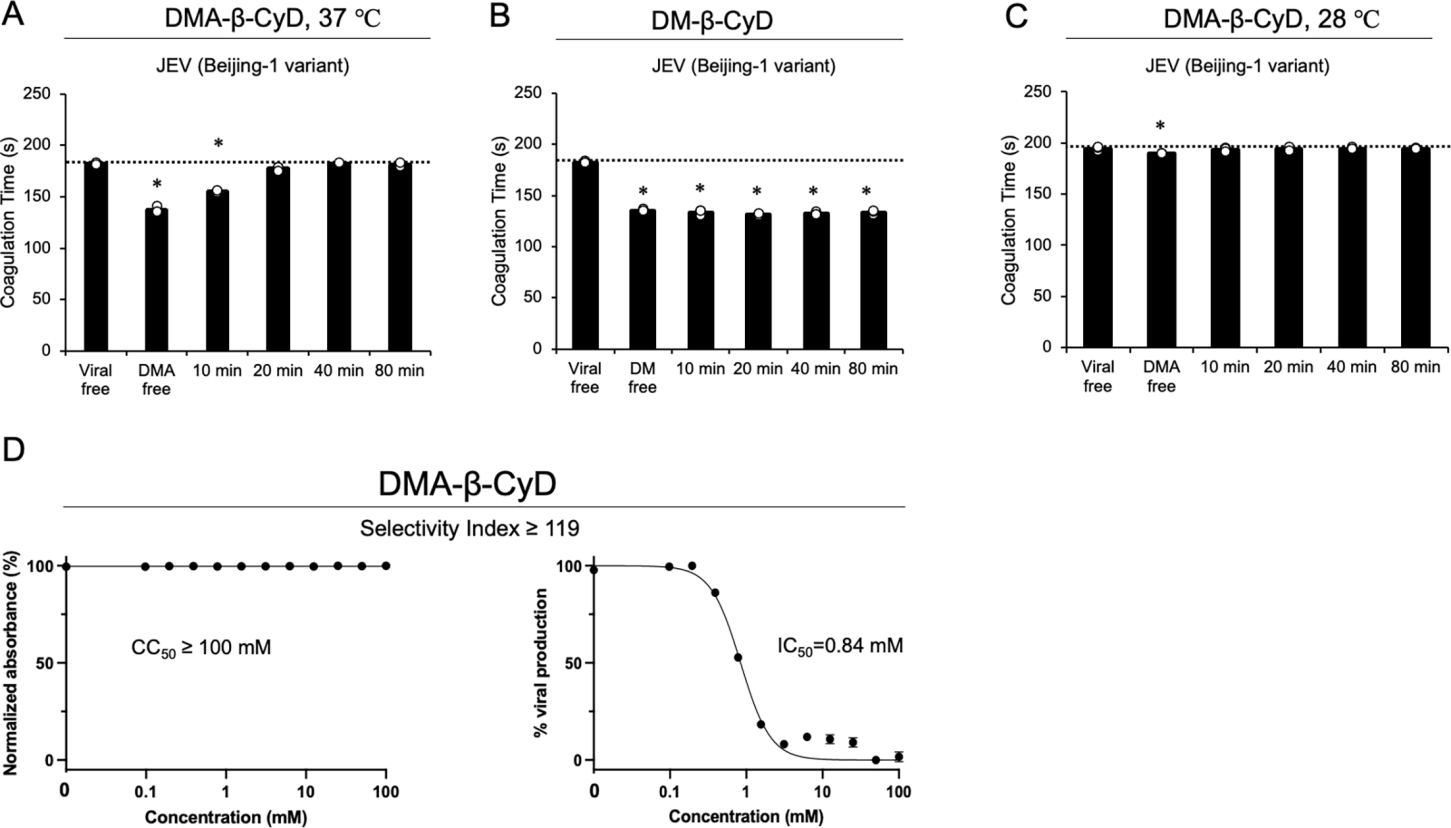
Evaluation of the anti-JEV activity of DMA-β-CyD. The JEV Beijing-1 variant, prepared in Vero cells, was treated with DMA-β-CyD (**A**) or DM-β-CyD (**B**) for the indicated times, followed by a clotting assay at 37°C. (**C**) The procoagulant effect of the JEV Beijing-1 variant at 28°C was analyzed. (**D**) Cytotoxicity and antiviral activity of DMA-β-CyD against the JEV Beijing-1 variant. Vero cells were exposed to JEV, and normalized absorbance (%) values were calculated by setting the absorbance at 450 nm of untreated, non-cytotoxic control cells to 100%. The percent viral production in the culture supernatants of Vero cells was determined using RT-qPCR. DMA-β-CyD was tested on at least three independent occasions. Data are shown as means ± SD. *P* values were determined using one-way ANOVA (Dunnett's test). * indicates a statistically significant difference in clotting time between groups untreated and treated with DMA-β-CyD or DM-β-CyD (*P* < 0.05). Data represent the means of at least three independent experiments, and error bars indicate SD.

To evaluate whether DMA-β-CyD inhibits infection by viruses other than SARS-CoV-2, we investigated its antiviral activity against JEV, a member of the *Flaviviridae* family. Similar to SARS-CoV-2, some studies indicated that JEV possesses PS on the surfaces of its particles and that PS mediates JEV infection (30, 31). It has been reported that SARS-CoV-2 has a low spike density, making the lipid bilayer more readily exposed (32). In contrast, cryo-EM analysis of JEV indicated that the surfaces of mature viral particles are almost entirely covered with the E protein, forming a generally smooth shell with limited “holes” (33). This finding suggests that JEV, whose surface is predominantly covered with the E protein, may have a reduced “area/accessibility” of PS compared with SARS-CoV-2, which possesses sparse spikes. This difference in spike density may impact their respective procoagulant capabilities. As shown in Fig. 6A, JEV exhibited weaker procoagulant activity than SARS-CoV-2, as expected; however, this activity was prolonged following treatment with DMA-β-CyD. In contrast, DM-β-CyD had no effect (Fig. 6B). These findings suggest that DMA-β-CyD may function by masking aminophospholipids present on the surfaces of JEV particles. Next, to examine whether JEV exhibits temperature-driven dynamic structural changes—often referred to as “viral breathing” within the *Flaviviridae* family—we assessed the procoagulant activity of JEV at 28°C and found that this activity was significantly attenuated. As expected, owing to the effects of enzymatic reactions associated with the temperature decrease, the average clotting time at 28°C in the control experiment was 195 s, approximately 13 s longer than the average clotting time at 37°C. Even with the DMA-β-CyD treatment of JEV, the clotting time remained similar to that of the control. This suggests that increasing the temperature from mosquito body temperature (28°C) to human body temperature (37°C) slightly relaxes the E protein lattice, resulting in greater exposure of PS in the viral lipid envelope, thereby allowing the PS interaction with DMA-β-CyD. Finally, we investigated the antiviral effect of DMA-β-CyD on JEV. As shown in Fig. 6D, the cytotoxicity of DMA-β-CyD in Vero cells yielded a CC₅₀ value of ≥100 mM, while the IC₅₀ value against JEV infection was 0.84 mM. Therefore, the SI of DMA-β-CyD was calculated to be ≥119. These results indicate that DMA-β-CyD inhibits the infection of not only SARS-CoV-2 but also JEV, demonstrating its inhibitory effect on viruses that possess aminophospholipids in their lipid envelopes.

## DISCUSSION

In this study, we found that DMA-β-CyD, among 19 cyclodextrin (CyD) derivatives used as pharmaceuticals and food additives, possesses anti-SARS-CoV-2 (IC_50_ = 0.28 mM, CC_50_ ≥ 100 mM, SI ≥ 357) and anti-JEV (IC_50_ = 0.84 mM, CC_50_ ≥ 100 mM, SI ≥ 119) activities. Bolland et al. (21), Bezerra et al. (22), and Raïch-Regué et al. (23) reported that β-CyD derivatives, such as methyl-β-CyD and HP-β-CyD, can effectively inhibit SARS-CoV-2 spike-protein-mediated entry and cell fusion by depleting cholesterol from the host cell membrane. However, for our screening system, we used host cells, such as Vero E6/TEMPRESS2, different from those in previous studies and did not use pseudoviruses for evaluation. This may have led to differences in the screening results. In particular, the lipid envelope composition of pseudoviruses differs from that of the original SARS-CoV-2, which may affect the results. Previously, DMA-β-CyD was reported to be highly safe for various eukaryotic cells compared with its precursor derivative, DM-β-CyD, owing to the presence of an acetyl group at position 3 of the molecule, which reduces the hydrophobicity of the lumen and loses its ability to encapsulate cholesterol (34). Furthermore, DMA-β-CyD interacts with lipid A, a component of lipopolysaccharide, an endotoxin component of Gram-negative bacteria (34), and can incorporate multiple hydrophobic alkyl chains (25). This study suggests that DMA-β-CyD may inhibit SARS-CoV-2 infection by both PS masking and reducing viral particle stability, without inhibiting direct binding of the SARS-CoV-2 spike protein to ACE2. Plasma clotting assays showed that SARS-CoV-2 and JEV particles decreased the plasma clotting time, but treatment with 10 mM DMA-β-CyD prolonged the clotting time, suggesting that DMA-β-CyD inhibits the procoagulant effects of PS and other components in the viral lipid envelope of these viruses. Because PS plays important roles in blood clotting and PS-receptor-mediated endocytosis (35, 36), DMA-β-CyD may mask PS in the lipid envelope and inhibit these functions. Furthermore, because DMA-β-CyD treatment inhibited SARS-CoV-2 entry into host cells, we investigated the stability of viral particles and found that DMA-β-CyD treatment reduced viral particle stability. This suggests that the binding efficiency of the spike protein to ACE2 itself was reduced, resulting in a lower viral infectivity titer. DMA-β-CyD may recognize the difference in lipid composition, where PS is more abundant on the outside of the viral lipid envelope than on the cell membrane (3). Although DMA-β-CyD was not toxic to red blood cells or cells at concentrations above 100 mM, further analysis is required to clarify the mechanism by which it reduces viral particle stability. We plan to explore the possibility that DMA-β-CyD reduces the infectivity titer by enveloping the palmitoylation sites that serve as spike protein anchors, ultimately inducing spike protein shedding. In addition, we intend to conduct a more thorough investigation into the mechanism by which DMA-β-CyD diminishes JEV infectivity, with a particular focus on the stability of the viral E protein on the viral particle surface. In summary, targeting the viral lipid envelope with DMA-β-CyD to reduce viral particle stability and infectivity could be an effective strategy against the emergence of mutant variants and new pandemic viruses. DMA-β-CyD is expected to become a novel approach for early responses to emerging infectious viruses.

## Data Availability

This study did not generate any new data sets. All data supporting the conclusions of this study are included in the article.
